# An Endoribonuclease Functionally Linked to Perinuclear mRNP Quality Control Associates with the Nuclear Pore Complexes

**DOI:** 10.1371/journal.pbio.1000008

**Published:** 2009-01-06

**Authors:** Michal Skružný, Claudia Schneider, Attila Rácz, Julan Weng, David Tollervey, Ed Hurt

**Affiliations:** 1 Biochemie-Zentrum der Universität Heidelberg (BZH), Heidelberg, Germany; 2 Wellcome Trust Centre for Cell Biology, University of Edinburgh, Edinburgh, Scotland; Emory University, United States of America

## Abstract

Nuclear mRNA export is a crucial step in eukaryotic gene expression, which is in yeast coupled to cotranscriptional messenger ribonucleoprotein particle (mRNP) assembly and surveillance. Several surveillance systems that monitor nuclear mRNP biogenesis and export have been described, but the mechanism by which the improper mRNPs are recognized and eliminated remains poorly understood. Here we report that the conserved PIN domain protein Swt1 is an RNA endonuclease that participates in quality control of nuclear mRNPs and can associate with the nuclear pore complex (NPC). Swt1 showed endoribonuclease activity in vitro that was inhibited by a point mutation in the predicted catalytic site. Swt1 lacked clear sequence specificity but showed a strong preference for single-stranded regions. Genetic interactions were found between Swt1 and the THO/TREX and TREX-2 complexes, and with components of the perinuclear mRNP surveillance system, Mlp1, Nup60, and Esc1. Inhibition of the nuclease activity of Swt1 increased the levels and cytoplasmic leakage of unspliced aberrant pre-mRNA, and induced robust nuclear poly(A)^+^ RNA accumulation in *mlp1*Δ and *esc1*Δ strains. Overexpression of Swt1 also caused strong nuclear poly(A)^+^ RNA accumulation. Swt1 is normally distributed throughout the nucleus and cytoplasm but becomes concentrated at nuclear pore complexes (NPCs) in the *nup133*Δ mutant, which causes NPC clustering and defects in mRNP export. The data suggest that Swt1 endoribonuclease might be transiently recruited to NPCs to initiate the degradation of defective pre-mRNPs or mRNPs trapped at nuclear periphery in order to avoid their cytoplasmic export and translation.

## Author Summary

Nuclear export of messenger RNA (mRNA) is a crucial step during eukaryotic gene expression. Newly synthesized precursor mRNAs are processed during synthesis, packaged into messenger ribonucleoprotein particles (mRNPs), and transported through the nuclear pore complex to the cytoplasm. To avoid nuclear export of aberrant transcripts and their translation in the cytoplasm, the quality of nuclear mRNPs is monitored by several surveillance systems. Here we show that the conserved protein Swt1 is an RNA endoribonuclease, an RNA-degrading enzyme, that becomes indispensable when factors involved in co-transcriptional mRNP assembly and mRNP quality control are mutated. We found that inactive Swt1 increases the levels and cytoplasmic leakage of aberrant, unprocessed precursor mRNA. Moreover, Swt1 accumulates at the nuclear pore complexes in the pore-clustering *nup133*Δ mutant. Thus, we speculate that the Swt1 endoribonuclease can be transiently recruited to the nuclear periphery to initiate the degradation of defective, pore-trapped pre-mRNPs in order to prevent their inappropriate cytoplasmic export.

## Abstract 3

When errors in messenger RNA processing or packaging occur along the path from the site of transcription to the nuclear pore complex, the conserved RNA-degrading enzyme Swt1 comes into the game.

## Introduction

Nuclear export of messenger RNA (mRNA) is a fundamental process of eukaryotic gene expression. In yeast, newly synthesized pre-mRNAs are cotranscriptionally assembled into messenger ribonucleoprotein particles (mRNPs) with the assistance of transcription-export (TREX) complexes. The THO/TREX (Tho2-Hpr1-Mft1-Thp2-Tex1-Sub2-Yra1) and TREX-2 (Sac3-Thp1-Sus1-Cdc31) complexes couple mRNA transcription with the recruitment of RNA binding proteins (e.g., Yra1, Npl3, and Nab2), and the conserved mRNA export receptor Mex67-Mtr2 (TAP-p15 in humans) [1,2]. In the absence of TREX or TREX-2 components, nascent mRNAs are not correctly assembled into mRNPs, which can lead to hybridization with the DNA template (R-loops formation), defective polyadenylation, and accumulation at the 3′ end of the gene [3–5]. As a result, export-incompetent mRNPs accumulate in the nucleus [6,7]. During maturation and transport, nuclear mRNPs are monitored by surveillance systems to prevent the export and subsequent translation of aberrant mRNAs (reviewed in [8,9]). In TREX mutants, imperfect mRNPs can be targeted and degraded by the nuclear exosome together with the TRAMP polyadenylation complex [10].

An important task of nuclear mRNA quality control is to avoid an export of intron-containing pre-mRNAs to the cytoplasm. The export of unspliced pre-mRNAs is rare in wild-type cells, whereas substantial leakage of pre-mRNA is seen in strains with defects in early splicing factors or for transcripts mutated in the 5′ splice site or the branch-point sequence [11–13]. Leakage of pre-mRNAs that lack any clear splicing defects was observed in cells lacking Mlp1 (a homolog of human TPR) or Nup60, two structural constituents of the nucleoplasmic basket of the nuclear pore complex (NPC) [14]. This suggested that Nup60-Mlp1 selectively traps intron-containing pre-mRNAs, and perhaps other aberrantly processed mRNAs, to prevent their exit from the nucleus [14,15]. More recently, Pml39, the binding partner of Mlp1, and Esc1, a protein of the nuclear periphery involved in the correct localization of the Nup60-Mlp1 complex, were shown to contribute to the function of Mlp1-Nup60 [16,17]. However, the mechanism by which the aberrant transcripts are recognized and eliminated remains unclear.

The yeast PIN domain protein Swt1/Yor166c (Synthetic lethal with TREX 1) was identified in a screen for synthetic lethality with the TREX subunit Hpr1, interacts functionally with the TREX complex and is required for optimal transcription rates [18]. The PIN (PilT N terminus) module is approximately 130 residues in length and adopts a fold similar to 5' exonucleases and FLAP endonucleases [19,20]. Typical features are four invariant acidic residues located in a negatively charged surface cleft, forming the predicted catalytic center [21]. These residues probably chelate a divalent ion and, together with two adjacent hydroxyl residues, are involved in the nucleophilic attack on the substrate phosphodiester bond. The PIN domain of human SMG6 exhibited endonuclease activity in vitro and is required for its function in nonsense-mediated mRNA decay (NMD) [22]. The yeast genome encodes eight PIN domain–containing proteins, including Nob1 and Utp24/Fcf1, that assist in endonucleolytic cleavages of rRNA precursors during ribosome biogenesis [23,24] and the Rrp44/Dis3 subunit of the exosome (C. Schneider and D. Tollervey, unpublished data). However, none of these yeast proteins has been demonstrated to function as an endonuclease.

Here, we report that the evolutionarily conserved protein Swt1 shows RNA endonuclease activity that is dependent on the PIN domain. Swt1 is essential in cells compromised for cotranscriptional mRNP formation or perinuclear mRNP quality control and can associate with NPCs. These findings reveal an important role for endonucleolytic cleavage in the process of nuclear mRNA surveillance.

## Results

### *SWT1* Functionally Overlaps with Factors Involved in Cotranscriptional mRNP Assembly and Perinuclear mRNP Quality Control

To identify factors that interact with TREX-2, a transcription-export complex, which functions at the inner side of the NPC, we performed a synthetic lethality (sl) screen with the *sac3*Δ strain. Among the candidates, we identified a mutant allele of the *SWT1*/*YOR166* gene. The genetic interaction was verified by absence of growth of a haploid *sac3*Δ *swt1*Δ strain, generated by plasmid loss (Figure S1). Other TREX-2 components Thp1 and Sus1 were either sl (*thp1*Δ) or showed strong synergistic interaction (*sus1*Δ) with *swt1*Δ, demonstrating an intimate functional interaction between Swt1 and TREX-2 complex (Table 1 and Figure S1).

**Table 1:**
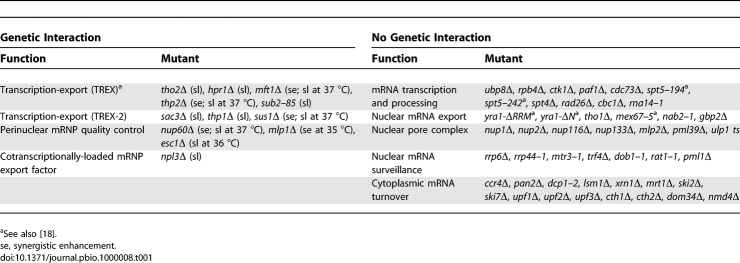
Genetic Interactions of *SWT1*

To extend these studies, we tested whether *SWT1* functionally overlaps with other factors involved in mRNP biogenesis and turnover. Strong genetic interactions were observed between *swt1*Δ and THO/TREX components (*tho2*Δ, *hpr1*Δ, *mft1*Δ, *thp2*Δ, and *sub2–85*) consistent with previous findings [18]. In contrast, no genetic interactions were detected between *swt1*Δ and the mRNA export receptor Mex67 or adaptor Yra1 [18]. In addition, we found an sl interaction between *swt1Δ* and *NPL3*, which encodes an mRNA binding protein with multiple roles in mRNP biogenesis [25,26] (Table 1 and Figure S1).

These studies also uncovered genetic interactions between *SWT1* and *MLP1*, *NUP60*, and *ESC1* (Table 1 and Figure S1). Mlp1 and Nup60 are NPC-associated factors that are thought to constitute the nuclear basket of NPC and participate in mRNP quality control before nuclear export [14]. Esc1 is a protein of the inner side of the nuclear membrane recently shown to affects the association of the Mlp1-Nup60 complex with the NPC [17].

Several factors implicated in various steps of mRNP production, export and surveillance did not interact genetically with *swt1Δ*, including subunits of the nuclear exosome (Rrp6 and Rrp44/Dis3), components of the TRAMP complex (Trf4 and Mtr4), the Rat1 5′-exonuclease, and factors involved in cytoplasmic mRNA turnover (see Table 1 for factors tested). These genetic analyses indicate that Swt1 interacts specifically with factors required for cotranscriptional assembly and perinuclear quality control of nuclear mRNPs.

### Swt1 Has Endoribonuclease Activity In Vitro

**Figure 1:**
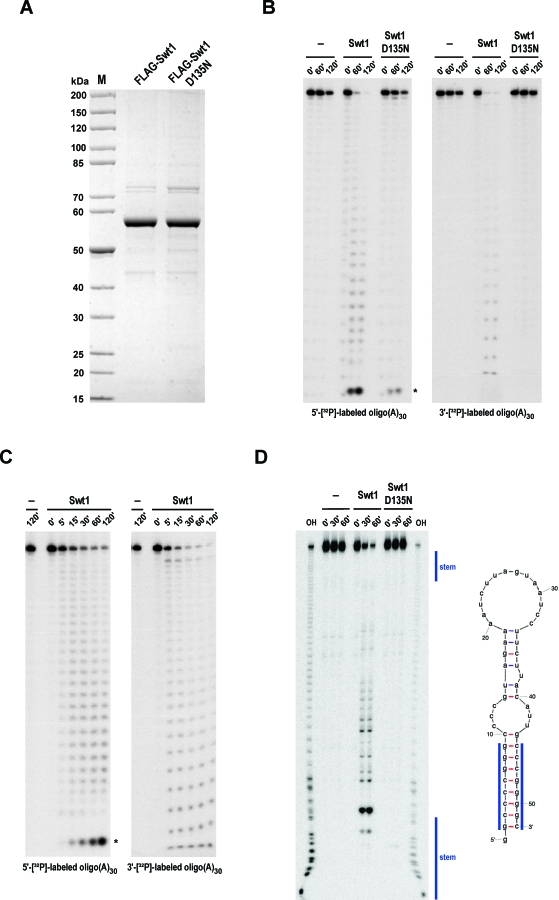
Swt1 Has Endonucleolytic Activity on Single-Stranded RNA In Vitro (A) Purified wild-type Swt1 and mutant Swt1-D135N proteins used for in vitro nuclease activity measurements. The indicated FLAG-tagged Swt1 proteins were affinity-purified from yeast and analyzed by SDS-PAGE and Coomassie staining. M, molecular weight marker. (B) Wild-type Swt1 or mutant Swt1-D135N protein (0.5 μg each) were incubated with 5′-[^32^P]-end–labeled (left panel) or 3′-[^32^P]-end–labeled (right panel) oligo(A)_30_ RNA for 0, 60, and 120 min at 30 °C. The reaction products were separated on a denaturing 12% polyacrylamide/8 M urea gel and visualized by autoradiography. RNA probes incubated without protein served as negative controls, indicated by the negative sign (−). The asterisk (*) denotes a low activity of a contaminating yeast exonuclease. (C) Time course of Swt1 RNase activity on 5′-[^32^P]-end–labeled (left panel) or 3′-[^32^P]-end–labeled (right panel) oligo(A)_30_ RNA. The assay was performed as described in (B), and aliquots were taken at the indicated time points. (D) Wild-type Swt1 or mutant Swt1-D135N were incubated with 5′-[^32^P]-end–labeled 52-nt RNA with a stable terminal stem for 0, 30, and 60 min at 30 °C, and the reaction was analyzed as outlined in (B). OH, a partial alkaline hydrolysis of the RNA probe used as a molecular weight ladder. The positions of the stem residues are indicated in blue on the right side of the gel and on the RNA secondary structure calculated by MFOLD.

The PIN domain of Swt1, which lies in the central region (residues 130–262), is evolutionarily conserved and contains all four conserved acidic residues implicated in catalysis (Figure S2). We therefore tested whether Swt1 purified from yeast has in vitro nuclease activity. We generated FLAG-tagged wild-type Swt1 and an Swt1 mutant expected to be catalytically inactive (Swt1-D135N; Asp 135 is predicted to be a residue of the catalytic center). These were expressed in yeast and purified by affinity chromatography (Figure 1A). The ability of these proteins to degrade single-stranded RNA in vitro was assessed using 5′- or 3′-radiolabeled oligo(A)_30_ substrates. Wild-type Swt1 efficiently degraded both 5′- and 3′-labeled oligo(A)_30_ RNA. In contrast, the Swt1-D135N mutant protein exhibited no activity on the 3′-labeled RNA substrate and only very weak activity on the 5′-labeled probe (Figure 1B). The residual activity on the 5′-labeled substrate could be due to a contaminating 3′-5′ exonuclease that is frequently observed in exonuclease assays (C. Schneider, unpublished data). The lack of degradation of the 3′-labeled substrate by this activity probably reflects protection by the presence of a 3′-phosphate on the [^32^P]pCp-labeled RNA. Time course experiments further showed that wild-type Swt1 degrades both 5′- and 3′-labeled substrates with very similar kinetics (Figure 1C), consistent with endoribonuclease activity.

To assess whether Swt1 functions as an endonuclease, a structured 52-nucleotide (nt) RNA substrate was used, which contains a stable stem (from the mouse 5.8S rRNA) that closes the 5'- and 3'-ends of the RNA (Figure 1D). The remaining part of the RNA (nt 11–43) is predicted to be at least partially single stranded. Wild-type Swt1, but not the Swt1-D135N mutant, efficiently cleaved the structured 52-nt RNA endonucleolytically, with a clear preference for single-stranded regions of the RNA substrate (Figure 1D). Taken together, the data show that Swt1 exhibits in vitro endonuclease activity without clear sequence specificity but with a preference for single-stranded RNA.

### Deletion of *SWT1* Induces Nuclear Poly(A)^+^ RNA Accumulation and Enhances Cytoplasmic Leakage of Splicing-Defective Pre-mRNAs in *mlp1*Δ and *nup60*Δ Mutants

**Figure 2:**
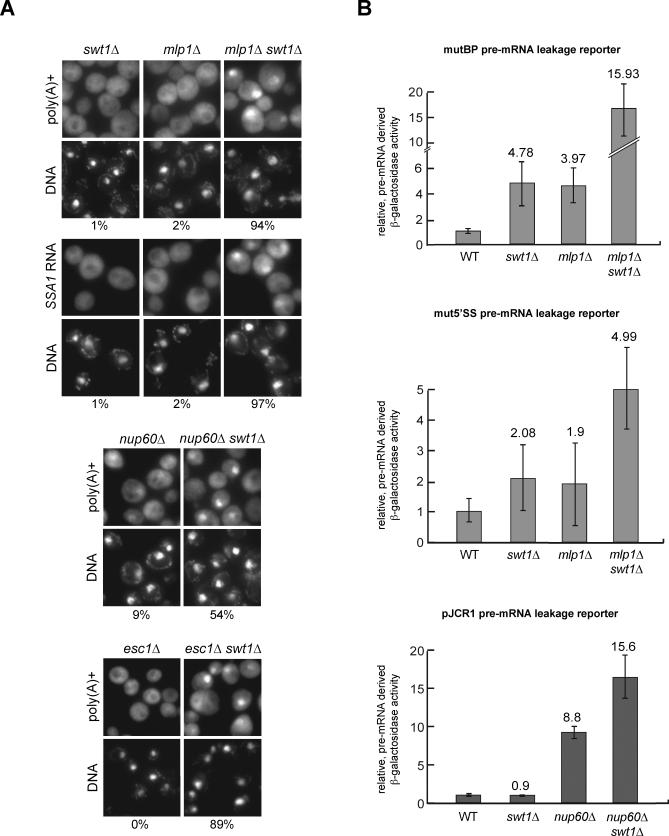
*SWT1* Is Involved in Mlp1-Nup60-Assisted Nuclear mRNP Quality Control (A) Disruption of *SWT1* induces or enhances the nuclear accumulation of poly(A)^+^ and *SSA1* RNA in *mlp1*Δ, *esc1*Δ, or *nup60*Δ strain, respectively. The indicated single- and double-mutant strains were grown at 30 °C (*nup60*Δ) or shifted to 37 °C for 90 min (*mlp1*Δ and *esc1*Δ), and the localization of poly(A)^+^ RNA and *SSA1* RNA was analyzed by in situ hybridization using Cy3-oligo(dT) and *SSA1*-specific probes, respectively. DNA was stained with DAPI. The percentage of the cells showing a nuclear accumulation of poly(A)^+^ RNA or *SSA1* mRNA, respectively, was calculated for *n* > 150 cells and indicated below each panel (*mlp1*Δ and *esc1*Δ strains). In the case of the *nup60*Δ strain, the percentage of cells with a significantly enhanced nuclear poly(A)^+^ signal (above 1.5-fold of the average intensity of the *nup60*Δ nuclear signal) is shown. (B) Deletion of *SWT1* induces a leakage of reporter pre-mRNAs to the cytoplasm and synergistically enhances the pre-mRNA leakage of *mlp1*Δ and *nup60*Δ cells. The leakage of unspliced *lacZ* pre-mRNA reporters that give rise to β-galactosidase activity was analyzed as described in Materials and Methods. The ratios between β-galactosidase activities derived from the reporter pre-mRNA and intronless control mRNA were calculated and shown relatively to the ratio of respective wild-type strain. The results of four independent experiments are shown. Error bars indicate standard deviations.

To further investigate the in vivo role of Swt1 in the context of transcription-coupled mRNA export and nuclear mRNP surveillance, we performed mRNA export and pre-mRNA leakage assays with the viable double mutants (Figure S1) carrying *swt1*Δ together with *thp2*Δ, *mft1*Δ (THO/TREX), *sus1*Δ (TREX-2), *nup60*Δ, *mlp1*Δ, and *esc1*Δ (perinuclear mRNP quality control). Deletion of *SWT1* significantly enhanced nuclear poly(A)^+^ RNA accumulation relative to the *thp2*Δ, *mft1*Δ, *sus1*Δ, and *nup60*Δ single mutants (Figures 2A, S3A and unpublished data). Moreover, *swt1*Δ induced nuclear accumulation of poly(A)^+^ RNA in the *mlp1*Δ and *esc1*Δ mutants, which was not detected in the single mutants (Figure 2A; see also [27,28]).

To assess whether the poly(A)^+^ RNA accumulation reflects the accumulation of nuclear mRNA, we tested a specific transcript, the heat-shock *SSA1* mRNA (Figure 2A). This mRNA was clearly accumulated in the nucleus of the *mpl1Δ swt1*Δ double mutant, but not in the corresponding single mutants. In contrast, no nuclear accumulation was observed when the nuclear export of tRNA was assayed (unpublished data). To quantify whether the levels of *SSA1* transcript were increased in the *mlp1*Δ *swt1*Δ double mutant, we measured the amount of *SSA1* mRNA before and after heat shock by real-time reverse-transcription polymerase chain reaction (RT-PCR). The *SSA1* levels were mildly increased in the single mutants, but a synergistic increase was observed in the *mlp1*Δ *swt1*Δ double mutant in comparison to wild-type strain (Figure S4).

To test whether the double mutants of *swt1Δ* and *mlp1Δ* or *nup60Δ* result in synergistic defects in pre-mRNP surveillance, we monitored cytoplasmic leakage of unspliced pre-mRNA reporters [11,12,14]. No leakage of the reporter with intact splicing signals (pJCR1) was observed in the *swt1Δ* strain, whereas the *nup60Δ* strain showed increased leakage of the unspliced pre-mRNA, which was synergistically enhanced in the *swt1*Δ *nup60*Δ double mutant (Figure 2B). Next, we tested whether pre-mRNA reporters with a mutation in the 5′ splicing site (mut5′SS) or in the branch-point site (mutBP) exhibit an increased cytoplasmic leakage in *swt1*Δ cells. Notably, leakage of the mutBP reporter was increased approximately 4-fold in the *swt1*Δ or *mlp1Δ* single mutants relative the wild type (Figure 2B; see also [14,16]). Cytoplasmic leakage of both mutant pre-mRNA reporters was again strongly synergistically enhanced in the *swt1Δ mlp1Δ* double mutant (Figure 2B).

We conclude that loss of Swt1 leads to increased cytoplasmic leakage of splicing-defective pre-mRNAs, which can be synergistically enhanced by mutations in the Mlp1-Nup60 complex. This suggests that Swt1 and Mlp1-Nup60 have distinct but overlapping roles in nuclear mRNP surveillance. At first sight, it appears contradictory that an increased pre-mRNA leakage goes along with nuclear accumulation of poly(A)^+^ RNA. However, these two effects are necessarily not mutually exclusive. When nuclear quality control is severely compromised (e.g., in *mlp1*Δ *swt1*Δ or *nup60*Δ *swt1*Δ cells), larger amounts of aberrant mRNPs could accumulate in the nucleus and hence have a higher chance to leak out.

### The Endonuclease Domain Is Essential for Swt1 Function at Physiological Expression Levels

**Figure 3:**
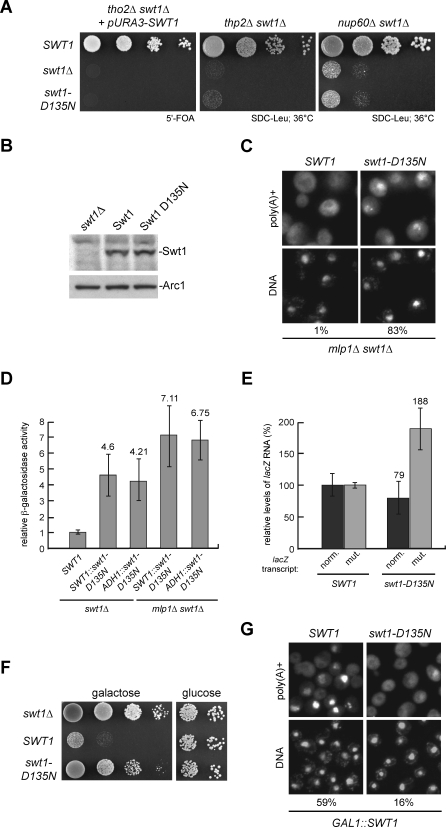
The Catalytically Inactive *swt1-D135N* Mutant Resembles the *swt1* Null Mutation In Vivo (A) The *swt1-D135N* mutant does not complement the synthetically lethal/enhanced phenotypes of the indicated *swt1*Δ double-mutant strains. Strains were transformed with wild-type *SWT1* or mutant *swt1-D135N*, and transformants were spotted in 10-fold serial dilutions on the indicated plates. The strains were incubated for 5 d at 30 °C (*tho2*Δ *swt1*Δ) or for 3 d at 36 °C (*thp2*Δ *swt1*Δ and *nup60*Δ *swt1*Δ). (B) The expression levels of wild-type and mutant Swt1 proteins are similar. Whole-cell lysates prepared from a *swt1*Δ strain or a *swt1*Δ strain expressing wild-type Swt1 or Swt1-D135N were analyzed by SDS-PAGE and western blotting using polyclonal anti-Swt1 antibodies. The detection of Arc1 protein by an anti-Arc1 polyclonal antibody was used as a loading control. (C) The Swt1-D135N mutant cannot rescue the poly(A)^+^ export defect of the *mlp1*Δ *swt1*Δ strain. *mlp1*Δ *swt1*Δ cells expressing wild-type Swt1 or Swt1-D135N were analyzed for poly(A)^+^ RNA localization as in Figure 2A or stained with DAPI to detect DNA. (D) Swt1-D135N-expressing cells show a nuclear pre-mRNA leakage phenotype. The *swt1*Δ and *mlp1*Δ *swt1*Δ strains were transformed with the indicated plasmids and assayed for the leakage of the *lacZ* pre-mRNA reporter (mutBP) as described in Figure 2B. The results of four independent experiments are shown. Error bars indicate standard deviations. (E) *Swt1-D135N* cells accumulate elevated levels of mutated *lacZ* pre-mRNA. Total RNA was isolated from the *swt1-D135N* and isogenic wild-type strain expressing the intron-containing *lacZ* reporter transcript (norm*.*, for normal) or the intron-containing *lacZ* transcript mutated in the branch-point splicing site (mut*.*, for mutated). The *lacZ* levels were quantified by real-time RT-PCR using the *GAL1* mRNA as a reference transcript. The results of four independent experiments are shown. Error bars indicate standard errors of the mean. (F) Overexpression of wild-type Swt1, but not overexpression of the catalytically inactive Swt1-D135N mutant, is toxic to the cells. *Swt1*Δ cells containing *GAL1::SWT1*, *GAL1::swt1-D135N*, or empty plasmid were spotted in 10-fold serial dilutions on plates containing either galactose or glucose and incubated for 3 d at 30 °C. Only the last two dilutions of glucose plate are shown. (G) Overexpression of wild-type Swt1, but not overexpression of the catalytically inactive Swt1-D135N mutant, induces strong nuclear accumulation of poly(A)^+^ RNA. The same strains as used in (F) were grown in raffinose-containing medium to early-log phase and then induced by 2% galactose for 5 h. The localization of poly(A)^+^ RNA was analyzed by in situ hybridization using a Cy3-oligo(dT) probe. DNA was stained with DAPI. The percentage of the cells showing an accumulation of nuclear poly(A)^+^ signal is shown (*n* > 150 cells).

In a previous study [18], Swt1 lacking the PIN (endonuclease) domain was shown to complement the lethality of *swt1Δ* in THO mutant strains. However, Swt1 is a low-abundance protein (see below), and the constructs were overexpressed from the relatively strong *NOP1* promoter. To assess whether the endonuclease activity of Swt1 is essential for complementation of *swt1Δ* phenotypes under physiological expression levels, we analyzed the catalytically inactive Swt1-D135N mutant expressed from the endogenous promoter (Figure 3A). Swt1-D135N and wild-type Swt1 were expressed at similar levels (Figure 3B), but Swt1-D135N failed to complement the growth defects of *swt1Δ* in any double-mutant combination (Figure 3A and unpublished data). Moreover, Swt1-D135N did not rescue the nuclear mRNA accumulation induced by *swt1Δ* in the background of the *mlp1*Δ, *nup60*Δ, *sus1*Δ, or *thp2*Δ mutations (Figure 3C and unpublished data). Finally, the nuclear leakage of the unspliced pre-mRNA reporter (mutBP) in Swt1-D135N–expressing cells (Figure 3D) was indistinguishable from the leakage observed in the *swt1*Δ strain (Figure 2B). Similar loss-of-function phenotypes were observed for the *swt1* truncation mutant lacking the entire PIN domain (Swt1ΔPIN) (Figure S5 and unpublished data).

However, Swt1-D135N, Swt1ΔPIN, and other partial deletion constructs could complement *swt1Δ* for growth in the *tho2Δ* background, when overexpressed under the control of the strong *ADH1* promoter (Figure S5). In contrast, other *swt1Δ* phenotypes, including nuclear pre-mRNA leakage and nuclear poly(A)^+^ RNA accumulation in *mlp1Δ swt1Δ* strains, could not be suppressed by overexpression of Swt1-D135N or Swt1ΔPIN mutants from the *ADH1* promoter (Figure 3D and unpublished data). Thus, under physiological expression levels, the endonuclease activity of Swt1 is essential for its function, whereas the requirement for catalytic activity can be partially relieved by overexpression of the inactive Swt1 protein.

### *Swt1-D135N* Cells Accumulate Elevated Levels of an Aberrant Pre-mRNA

To assess possible roles for the endonuclease activity of Swt1 in the degradation of defective mRNPs, we determined whether an “aberrant” transcript is stabilized in *swt1-D135N* cells relative to the otherwise isogenic wild type. As an abnormal transcript, we used the intron-containing *lacZ* construct (expressed from mutBP plasmid; see above) that was used in the pre-mRNA leakage assays. This harbors a mutation in the splicing branch-point site that efficiently blocks pre-mRNA splicing [12]. The wild-type intron-containing *lacZ* transcript encoded by plasmid pJCR51 was used as a control pre-mRNA. Accumulation of the aberrant transcript was substantially elevated in *swt1-D135N* cells relative to wild-type strain (mut. bars in Figure 3E), whereas the levels of normally processed *lacZ* mRNA was unchanged between the *swt1-D135N* and wild-type strains (norm. bars in Figure 3E). These results suggest that the endoribonuclease activity of Swt1 participates in the selective elimination of defective mRNA transcripts.

### Swt1 Overexpression Is Toxic and Induces a Strong Nuclear Accumulation of Pre-mRNA

As Swt1 participates in nuclear mRNP surveillance, its expression and/or targeting may be subject to regulation. Quantitative western blot analysis using anti-Swt1 antibodies indicated that in vivo levels of Swt1 endonuclease are very low (∼200–300 molecules per cell) (unpublished data; see also [18]), which might be important to avoid inappropriate RNA degradation. In a large-scale analysis, Yor166/Swt1 was reported to be toxic following overexpression by the Tet-off system [29]. Consistent with this finding, a dominant-lethal phenotype was induced by overexpression of wild-type Swt1 but not of catalytically inactive mutant Swt1-D135N from the *GAL1* promoter (Figure 3F). The majority of Swt1 overexpressing cells showed strong nuclear accumulation of both poly(A)^+^ RNA (Figure 3E) and *SSA1* mRNA, whereas tRNA was normally exported (Figure S3B and unpublished data). Notably, when the localization of intron-containing/intronless *lacZ* reporter constructs was analyzed, the nuclear accumulation of an intron-containing pre-mRNA reporter was stronger than for the intronless mRNA (Figure S3C), consistent with a defect in pre-mRNA surveillance. These analyses demonstrate that overexpression of catalytically active Swt1 is toxic and interferes with mRNA export and/or pre-mRNA surveillance.

### Swt1 Associates with the Nuclear Pore Complexes in *nup133*Δ Cells

**Figure 4:**
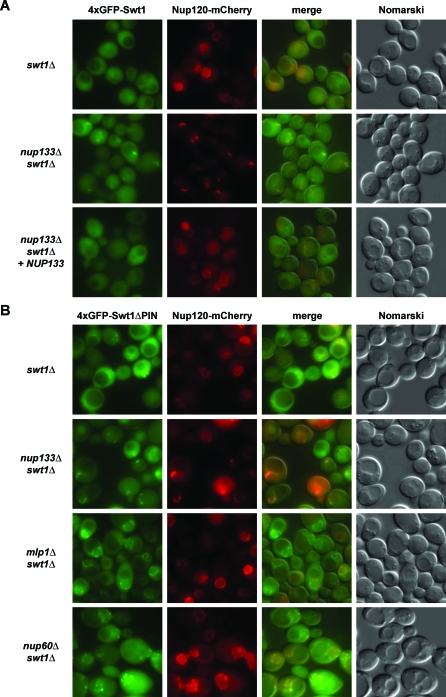
Swt1 Associates with Nuclear Pore Complexes in the Nucleoporin Mutant *nup133*Δ (A) 4xGFP-Swt1 (green) or (B) 4xGFP-Swt1ΔPIN (green) were coexpressed with Nup120-mCherry (a marker for NPCs; red) in the indicated strains (*swt1*Δ, *nup133*Δ *swt1*Δ, *nup133*Δ *swt1*Δ + *NUP133* [expressed from a centromeric plasmid], *mlp1*Δ *swt1*Δ, and *nup60*Δ *swt1*Δ) and analyzed by fluorescence microscopy. Cells were also viewed by Nomarski optics.

Subcellular fractionation previously indicated that TAP-tagged Swt1 is located in the nucleus [18]. The exact location of Swt1 could not be determined, however, because the abundance of green fluorescent protein (GFP)- or immuno-labeled Swt1 expressed from the endogenous promoter was too low to be visualized (unpublished data and [18]). The attachment of multiple GFP tags can allow the detection of low-abundance proteins (N. Daigle, A. Bancaud, and J. Ellenberg, unpublished data). We therefore generated a functional 4xGFP-Swt1 construct expressed from *SWT1* endogenous promoter, which complemented the double-deletion strains *swt1*Δ *mlp1*Δ, *swt1*Δ *nup60*Δ, or *swt1*Δ *tho2*Δ (Figure S6 and unpublished data). When analyzed in the *swt1*Δ strain, 4xGFP-Swt1 was localized in both the nucleus and cytoplasm, with a slight nuclear enrichment in some cells (Figure 4A). When expressed in the *nup133*Δ nuclear pore-clustering mutant [30], 4xGFP-Swt1 exhibited significant coclustering with NPCs at the nuclear periphery, which was lost upon complementation of the *nup133*Δ strain by plasmid-derived *NUP133* (Figure 4A). When a 4xGFP-Swt1 construct lacking the catalytic PIN domain (4xGFP-Swt1ΔPIN) was expressed in *swt1*Δ cells, it was concentrated in a few spots. These were always close to the NPCs, as revealed by covisualization of Nup120-mCherry (Figure 4B), and 4xGFP-Swt1ΔPIN also associated with NPCs in *nup133*Δ cells. Targeting of Swt1ΔPIN to the NPCs did not depend on Mlp1, Nup60, or Esc1. Instead, 4xGFP-Swt1ΔPIN showed more distinct and punctuate nuclear envelope staining in the *mlp1*Δ, *nup60*Δ, and *esc1*Δ strains than in the corresponding isogenic wild-type strains (Figure 4B and unpublished data). The nucleocytoplasmic localization of full-length 4xGFP-Swt1 in the *mlp1*Δ, *nup60*Δ, and *esc1*Δ cells was not altered (unpublished data). Together with the genetic data, these observations indicate that Swt1 interacts with the NPCs.

## Discussion

We show in this study that Swt1 is an RNA endonuclease that can associate with NPCs and participates in the systems that control the quality of nuclear mRNPs prior to export to the cytoplasm. The endonuclease activity of the PIN domain of Swt1 is crucial for its in vivo function at physiological (i.e., low) expression levels. Heavily overexpressed Swt1 constructs lacking the PIN domain could fulfill some of the in vivo functions of Swt1, whereas overexpression of active Swt1 was lethal. This indicates that Swt1 functions both as a nuclease and as a protein component of mRNP surveillance complexes.

To avoid the cytoplasmic translation of defective mRNPs into a potentially deleterious proteins, mRNP biogenesis and surveillance systems cooperate in the nucleus. The endoribonuclease activity of Swt1 is essential when the function of complexes that cotranscriptionally form and check the nuclear mRNPs is impaired, suggesting that Swt1 could be involved in degrading the mRNA component of aberrant nuclear mRNPs. An endonuclease activity could be important because transcriptionally trapped mRNPs, export-incompetent mRNPs, or pre-mRNPs incapable of splicing all lack accessible RNA ends that would allow degradation by the nuclear 5′-exonuclease Rat1 or the 3′-exonuclease activity of the exosome. Endonuclease cleavage of defective mRNA and mRNPs by Swt1 could generate free mRNA ends for further exonucleolytic degradation. Consistent with this model, an aberrant pre-mRNA reporter construct that cannot be spliced was selectively accumulated in the catalytically inactive Swt1-D135N mutant, and loss of Swt1 from the *mlp1Δ* strain stabilized the nuclear-restricted *SSA1* mRNA.

High levels of Swt1 were toxic and induced strong nuclear poly(A)^+^ RNA accumulation. Among the several possibilities, which could explain this phenotype, overexpressed Swt1 could induce erroneous RNA cleavage in properly formed RNP complexes. We estimate the number of Swt1 molecules in the cell to be only about 200–300 copies, and this low level may be important to suppress off-target RNA cleavage. The nuclear accumulation of an intron-containing reporter pre-mRNA induced by Swt1 overexpression was stronger than for the corresponding intronless pre-mRNA, consistent with a defect in pre-mRNA surveillance. Notably, overexpression of Mlp1, but not deletion of its gene, also caused nuclear accumulation of intron-containing pre-mRNAs [14,28], consistent with the model that Swt1 and Mlp1 function together in this pathway.

Our data are consistent with the previously suggested role of Swt1 in TREX-assisted mRNA transcription [18]. TREX/TREX-2 complexes are required for a cotranscriptional mRNP assembly, and failure in their function leads to formation of aberrant mRNP structures, which impede RNAPII transcription [3–5,9]. We predict that these defective mRNP particles represent important substrates for endonuclease degradation by Swt1. Consistent with this model, defective and export-incompetent mRNPs formed in TREX mutants accumulate in association with the NPCs [9,31], where Swt1 can also be detected.

In wild-type cells, Swt1 was distributed between the nucleus and cytoplasm, and hence could potentially detect the formation of defective pre-mRNPs all along the mRNP biogenesis pathway. However, Swt1 was accumulated at the NPCs when these were clustered in the *nup133*Δ mutant [30]. This NPC localization of Swt1 cannot be correlated to an impaired mRNA export (*nup133*Δ cells also exhibit an mRNA export defect), as 4xGFP-Swt1 did not accumulate at the nuclear envelope in the *mex67–5* mRNA export mutant (unpublished data). Moreover, Swt1 lacking its endonuclease domain became concentrated in perinuclear spots even in cells with a normal complement of nucleoporins. We speculate that Swt1 is transiently recruited to the NPCs via interactions with nucleoporins and/or with export-incompetent pre-mRNPs blocked at the NPCs, and function there to prevent inappropriate pre-mRNA export.

Analyses of nuclear degradation of defective RNAs in yeast have largely concentrated on the activities of the 5′- and 3′-exonucleases. This work demonstrates that endonuclease cleavage is also likely to play an important role in nuclear RNA surveillance.

## Materials and Methods

### Yeast strains, plasmids, and genetic manipulations.

Yeast strains used in this study are listed in Table S1; plasmid constructs are described in Table S2. Site-directed mutagenesis of the *SWT1* gene to create the *swt1-D135N* mutant was performed by fusion PCR [32]. The synthetic lethality screen with the *sac3*Δ strain was performed as previously described [33].

### In vitro nuclease activity assay.

For in vitro measurements of Swt1 nuclease activity, the FLAG-Swt1 wild-type and D135N mutant protein were expressed from plasmids pADH111-FLAG-*SWT1* or pADH111-FLAG-*swt1*-*D135N* (*ADH1* promoter) and purified by anti-FLAG affinity chromatography. Briefly, 4 l of yeast culture were harvested at an optical density at 600 nm (OD_600_) of approximately 4.0, resuspended in FLAG buffer (50 mM Tris-HCl [pH 7.5], 200 mM NaCl, 0.5 mM DTT, 0.05% NP-40) to a final volume of 50 ml, lysed by bead-beating (Pulverissete 6; Fritsch) and incubated with 120 μl of anti-FLAG M2 agarose (Sigma) for 120 min at 4 °C. Anti-FLAG slurry was washed by 750 volumes of FLAG buffer, and the bound material was eluted with FLAG peptide according to the manufacturer's instructions (Sigma) followed by concentration and buffer exchange.

The analyses of nuclease activity of the purified proteins on 5′-end– or 3′-end–radiolabeled RNAs were performed at 30 °C in 15 mM Tris-HCl (pH 7.6), 75 mM NaCl, 2 mM DTT, 100 μg/ml BSA, 0.8 U/μl RNasin, 4.5% glycerol, 0.05% NP-40, 0.3 μM Escherichia coli tRNA, and 7.5 mM MnCl_2_. Prior to addition of 20–30 fmoles of the labeled RNA substrate, the 20 or 30 μl reactions containing 10 pmoles of protein were pre-incubated for 15 min at 30 °C. Subsequently, 4-μl aliquots were taken at the time points indicated in Figure 1, and the reaction was stopped by the addition of formamide-containing RNA loading buffer. Reaction products were resolved on a denaturing 12% polyacrylamide/8 M urea gel and visualized by autoradiography. 5′-[^32^P]– and 3′-[^32^P]–labeled RNA substrates (Dharmacon) were labeled and gel-purified as described [34].

### Pre-mRNA leakage assays.

At least four independent experiments were performed for each strain containing an indicated *lacZ* pre-mRNA leakage reporter (pJCR1, pmut5′SS, and pmutBP) or intronless *lacZ* control (pLGSD5). Transformants were pooled and grown overnight in selective raffinose medium. Cells were diluted, grown to mid-log phase and the expression of reporter was induced by 2% galactose for 150 min. β-Galactosidase assays were performed in triplicate for each sample according to the published protocol [11] with the modifications described in [16].

### Analysis of *SSA1* and *lacZ* RNA levels.

To induce *SSA1* expression, heat shock (42 °C for 15 min) was applied to exponentially grown cultures by the addition of an equal volume of prewarmed medium. The expression of *lacZ* pre-mRNA reporters (pmutBP or pJCR51) was induced by an addition of galactose (2% final concentration) to early-log phase cultures and their subsequent incubation in minimal galactose-raffinose medium for 6 h. Cells were lysed by glass beads and vortexing, and total RNA was isolated with the RNeasy Mini Kit (QIAGEN). RNA was reverse transcribed using the QuatiTect Reverse Transcription Kit (QIAGEN), and levels of specific RNA transcript were measured by quantitative real-time PCR using the ABI Prism 7000 system (Applied Biosystems) and ABsolute QPCR ROX Mix (ABgene). Each cDNA sample, as well as controls without reverse transcriptase, was amplified in triplicate with the primer and probe sets described in Table S3. The levels of *SSA1* and *lacZ* RNA were normalized to *RPB4* and *GAL1* mRNA levels, respectively, which were found to be unchanged under the tested conditions.

### Miscellaneous.

Fluorescence in situ hybridization (FISH) using oligo(dT), *SSA1* transcript-, tRNA-, and *lacZ*-specific Cy3-labeled probes was performed as described [33,35,14]. The localization of the 4xGFP-Swt1, 4xGFP-Swt1ΔPIN, and Nup120-mCherry fusions was analyzed in a Zeiss Axio Imager.Z1 microscope equipped with AxioVision 4.6.3 software. The expression of Swt1 was analyzed by immunoblotting using rabbit polyclonal anti-Swt1 serum generated against the recombinant Swt1 (PickCell Laboratories) and anti-rabbit HRP-conjugate as a secondary antibody (BIO-RAD). The expression of FLAG-tagged Swt1 protein variants was analyzed by anti-FLAG-HRP conjugate (Sigma).

## Supporting Information

Figure S1 Genetic Interactions between *SWT1* and Factors of Cotranscriptional mRNP Assembly and Perinuclear mRNP Quality Control The indicated wild-type, single-mutant, and double-mutant strains were spotted in 10-fold serial dilutions on 5'-fluoroorotic acid (FOA)-containing plates in the case of a synthetic lethal interaction (left panel) and on synthetic dextrose complete (SDC)-Leu plates after 5'-FOA shuffling in the case of an synergistically enhanced growth defect (middle and right panels). Plates were incubated for 5 d at 30 °C (left panel) or 3 d at 30 °C or 36 °C (middle and right panels).
(2.23 MB PDF)

Figure S2 Sequence Comparison of PIN Domains of Swt1 and SMG6/EST1A Proteins The PIN domain sequences of Swt1 proteins from Saccharomyces cerevisiae (Swt1; Yor166c), Schizosaccharomyces pombe (SpSwt1; Spcc24b10.15p), Drosophila melanogaster (DmSwt1; CG7206-PB), and Homo sapiens (HsSwt1; C1orf26) were aligned to the PIN domains of *Drosophila* and human SMG6/EST1A proteins (DmSmg6, HsSmg6) by ClustalW. Asterisks indicate the conserved acidic residues implicated in the PIN domain nuclease activity. The positions of respective Swt1 residues are indicated.
(88 KB PDF)

Figure S3 Nuclear mRNA Accumulation after the Swt1 Deletion and Overexpression (A) Deletion of *SWT1* enhances the nuclear accumulation of poly(A)^+^ RNA of TREX-2 and TREX mutants. The indicated single- and double-mutant strains were shifted to 37 °C for 90 min, and the localization of poly(A)^+^ RNA was analyzed by in situ hybridization using a Cy3-oligo(dT) probe. DNA was stained with DAPI. The percentage of the cells showing a significantly enhanced nuclear poly(A)^+^ signal (above 1.5-fold of the average nuclear signal intensity of the respective single-mutant strain) is shown (*n* > 150 cells).
(B) Overexpression of Swt1 triggers the nuclear accumulation of *SSA1* mRNA. *Swt1*Δ cells or *swt1*Δ cells expressing *GAL1::SWT1* were grown in selective raffinose medium to an early-log phase, induced by 2% galactose for 5 h, and the localization of *SSA1* mRNA was detected by in situ hybridization using a *SSA1*-specific, Cy3-labeled probe. DNA was stained with DAPI. The percentage of the cells showing a nuclear accumulation of *SSA1* signal was calculated (*n* > 150 cells) and is depicted below.
(C) Overexpression of Swt1 causes the nuclear accumulation of *lacZ* mRNA reporters. Intronless (pLGSD5) or intron-containing (pJCR51) *lacZ* mRNA was expressed in wild-type, *swt1*Δ, or *GAL1::SWT1* strain, and its distribution was analyzed as in (B) using a *lacZ*-specific probe. In each case, at least 300 cells were inspected, and the percentage of the cells with the nuclear accumulation of *lacZ* signal is indicated.
(2.30 MB PDF)

Figure S4 *mlp1Δ swt1Δ* Double Disruption Leads to Elevated Cellular Levels of *SSA1* Transcript after the Heat Shock Total RNA was isolated from the indicated mid-log phase cultures before (25 °C) or after the heat shock to 42 °C for 15 min. The *SSA1* levels were quantified by real-time RT-PCR analysis using *RPB4* mRNA as a reference transcript. The representative results of two independent experiments are shown.
(65 KB PDF)

Figure S5 Deletion Analysis of Swt1 (A) Scheme of Swt1 constructs used for the deletion analysis. Numbers indicate the respective amino acid position along the Swt1 protein.
(B) Deletion analysis of Swt1. The *tho2Δ swt1Δ* shuffle train was transformed with indicated *SWT1* deletion constructs expressed from the endogenous *SWT1* or from a strong *ADH1* promoter and the complementation capability was tested by growth on 5'-FOA plates at 30 °C for 5 d.
(515 KB PDF)

Figure S6 Characterization of 4xGFP-Swt1 and 4xGFP-Swt1ΔPIN Fusion Proteins (A) The 4xGFP-Swt1 fusion complements the synthetically enhanced phenotypes of *mlp*Δ *swt1*Δ and *nup60*Δ *swt1*Δ double-mutant strains. Strains were transformed with plasmid YCplac111-FLAG-4xGFP-*SWT1*, YCplac111-FLAG-*SWT1*, or with empty plasmid, and transformants were spotted in 10-fold serial dilutions on the SDC-Leu plates. The strains were incubated for 5 d at the indicated temperatures.
(B) The comparison of expression levels of Swt1, Swt1ΔPIN, 4xGFP-Swt1, and 4xGFP-Swt1ΔPIN proteins. Whole-cell lysates were prepared from the *swt1*Δ or *mlp*Δ *swt1*Δ strains expressing the indicated constructs from centromeric YCplac111 plasmid and analyzed by SDS-PAGE and western blotting using a monoclonal anti-FLAG antibody. The asterisk denotes a protein in the cell lysate cross-reacting with the anti-FLAG conjugate.
(457 KB PDF)

Table S1 Yeast Strains Used in This Study (73 KB DOC)

Table S2 Plasmids Used in This Study (69 KB DOC)

Table S3 Real-Time RT-PCR Oligonucleotides Used in This Study (35 KB DOC)

## Abbreviations

GFP: green fluorescent protein
mRNP: messenger ribonucleoprotein particle
NPC: nuclear pore complex
RT-PCR: reverse-transcription polymerase chain reaction
sl: synthetic lethality
TREX: transcription-export

## References

[pbio-1000008-b001] Köhler A, Hurt E (2007). Exporting RNA from the nucleus to the cytoplasm. Nat Rev Mol Cell Biol.

[pbio-1000008-b002] Rougemaille M, Villa T, Gudipati RK, Libri D (2008). mRNA journey to the cytoplasm: attire required. Biol Cell.

[pbio-1000008-b003] Huertas P, Aguilera A (2003). Cotranscriptionally formed DNA:RNA hybrids mediate transcription elongation impairment and transcription-associated recombination. Mol Cell.

[pbio-1000008-b004] Gonzales-Aguilera C, Tous C, Gomez-Gonzales B, Huertas P, Luna R (2008). The THP1-SAC3-SUS1-CDC31 Complex works in transcription elongation-mRNA export preventing RNA-mediated genome instability. Mol Biol Cell.

[pbio-1000008-b005] Saguez C, Schmid M, Olesen JR, Ghazy MA, Qu X (2008). Nuclear mRNA surveillance in THO/sub2 mutants is triggered by inefficient polyadenylation. Mol Cell.

[pbio-1000008-b006] Strässer K, Masuda S, Mason P, Pfannstiel J, Oppizzi M (2002). TREX is a conserved complex coupling transcription with messenger RNA export. Nature.

[pbio-1000008-b007] Fischer T, Strässer K, Racz A, Rodriguez-Navarro S, Oppizzi M (2002). The mRNA export machinery requires the novel Sac3p-Thp1p complex to dock at the nucleoplasmic entrance of the nuclear pores. EMBO J.

[pbio-1000008-b008] Fasken MB, Corbett AH (2005). Process or perish: quality control in mRNA biogenesis. Nat Struct Mol Biol.

[pbio-1000008-b009] Villa T, Rougemaille M, Libri D (2008). Nuclear quality control of RNA polymerase II ribonucleoproteins in yeast: tilting the balance to shape the transcriptome. Biochim Biophys Acta.

[pbio-1000008-b010] Rougemaille M, Gudipati RK, Olesen JR, Thomsen R, Seraphin B (2007). Dissecting mechanisms of nuclear mRNA surveillance in THO/sub2 complex mutants. EMBO J.

[pbio-1000008-b011] Legrain P, Rosbash M (1989). Some cis- and trans-acting mutants for splicing target pre-mRNA to the cytoplasm. Cell.

[pbio-1000008-b012] Rain JC, Legrain P (1997). In vivo commitment to splicing in yeast involves the nucleotide upstream from the branch site conserved sequence and the Mud2 protein. EMBO J.

[pbio-1000008-b013] Rutz B, Séraphin B (2000). A dual role for BBP/ScSF1 in nuclear pre-mRNA retention and splicing. EMBO J.

[pbio-1000008-b014] Galy V, Gadal O, Fromont-Racine M, Romano A, Jacquier A, Nehrbass U (2004). Nuclear retention of unspliced mRNAs in yeast is mediated by perinuclear Mlp1. Cell.

[pbio-1000008-b015] Vinciguerra P, Iglesias N, Camblong J, Zenklusen D, Stutz F (2005). Perinuclear Mlp proteins downregulate gene expression in response to a defect in mRNA export. EMBO J.

[pbio-1000008-b016] Palancade B, Zuccolo M, Loeillet S, Nicolas A, Doye V (2005). Pml39, a novel protein of the nuclear periphery required for nuclear retention of improper messenger ribonucleoparticles. Mol Biol Cell.

[pbio-1000008-b017] Lewis A, Felberbaum R, Hochstrasser M (2007). A nuclear envelope protein linking nuclear pore basket assembly, SUMO protease regulation, and mRNA surveillance. J Cell Biol.

[pbio-1000008-b018] Röther S, Clausing E, Kieser A, Strässer K (2006). Swt1, a novel yeast protein, functions in transcription. J Biol Chem.

[pbio-1000008-b019] Clissold PM, Ponting CP (2000). PIN domains in nonsense-mediated mRNA decay and RNAi. Curr Biol.

[pbio-1000008-b020] Anantharaman V, Aravind L (2006). The NYN domains: novel predicted RNAses with a PIN domain-like fold. RNA Biol.

[pbio-1000008-b021] Jeyakanthan J, Inagaki E, Kuroishi C, Tahirov TH (2005). Structure of PIN-domain protein PH0500 from Pyrococcus horikoshii. Acta Crystallograph Sect F Struct Biol Cryst Commun.

[pbio-1000008-b022] Glavan F, Behm-Ansmant I, Izaurralde E, Conti E (2006). Structures of the PIN domains of SMG6 and SMG5 reveal a nuclease within the mRNA surveillance complex. EMBO J.

[pbio-1000008-b023] Fatica A, Tollervey D, Dlakic M (2004). PIN domain of Nob1p is required for D-site cleavage in 20S pre-rRNA. RNA.

[pbio-1000008-b024] Bleichert F, Granneman S, Osheim YN, Beyer AL, Baserga SJ (2006). The PINc domain protein Utp24, a putative nuclease, is required for the early cleavage steps in 18S rRNA maturation. Proc Natl Acad Sci U S A.

[pbio-1000008-b025] Lei EP, Krebber H, Silver PA (2001). Messenger RNAs are recruited for nuclear export during transcription. Genes Dev.

[pbio-1000008-b026] Gilbert W, Guthrie C (2004). The Glc7p nuclear phosphatase promotes mRNA export by facilitating association of Mex67p with mRNA. Mol Cell.

[pbio-1000008-b027] Strambio-de-Castillia C, Blobel G, Rout MP (1999). Proteins connecting the nuclear pore complex with the nuclear interior. J Cell Biol.

[pbio-1000008-b028] Kosova B, Panté N, Rollenhagen C, Podtelejnikov A, Mann M (2000). Mlp2p, a component of nuclear pore attached intranuclear filaments, associates with Nic96p. J Biol Chem.

[pbio-1000008-b029] Boyer J, Badis G, Fairhead C, Talla E, Hantraye F (2004). Large-scale exploration of growth inhibition caused by overexpression of genomic fragments in Saccharomyces cerevisiae. Genome Biol.

[pbio-1000008-b030] Doye V, Wepf R, Hurt EC (1994). A novel nuclear pore protein Nup133p with distinct roles in poly(A)+ RNA transport and nuclear pore distribution. EMBO J.

[pbio-1000008-b031] Rougemaille M, Dieppois G, Kisseleva-Romanova E, Gudipati RK, Lemoine S (2008). THO/Sub2p functions to coordinates 3′-end processing with gene-nuclear pore association. Cell.

[pbio-1000008-b032] Ho SN, Hunt HD, Horton RM, Pullen JK, Pease LR (1989). Site-directed mutagenesis by overlap extension using the polymerase chain reaction. Gene.

[pbio-1000008-b033] Strässer K, Hurt E (2001). Splicing factor Sub2p is required for nuclear mRNA export through its interaction with Yra1p. Nature.

[pbio-1000008-b034] Mitchell P, Petfalski E, Tollervey D (1996). The 3' end of yeast 5.8S rRNA is generated by an exonuclease processing mechanism. Genes Dev.

[pbio-1000008-b035] Long RM, Elliott DJ, Stutz F, Rosbash M, Singer RH (1995). Spatial consequences of defective processing of specific yeast mRNAs revealed by fluorescent in situ hybridization. RNA.

