# Supplementary material for: An Endoribonuclease Functionally Linked to Perinuclear mRNP Quality Control Associates with the Nuclear Pore Complexes
Source: PLoS Biol. 2009 Jan 6;7(1):e1000008. doi: 10.1371/journal.pbio.1000008 (PMC2613419; doi:10.1371/journal.pbio.1000008)
Supplement: Supplementary file 1 [file pbio.1000008.sg001.pdf]

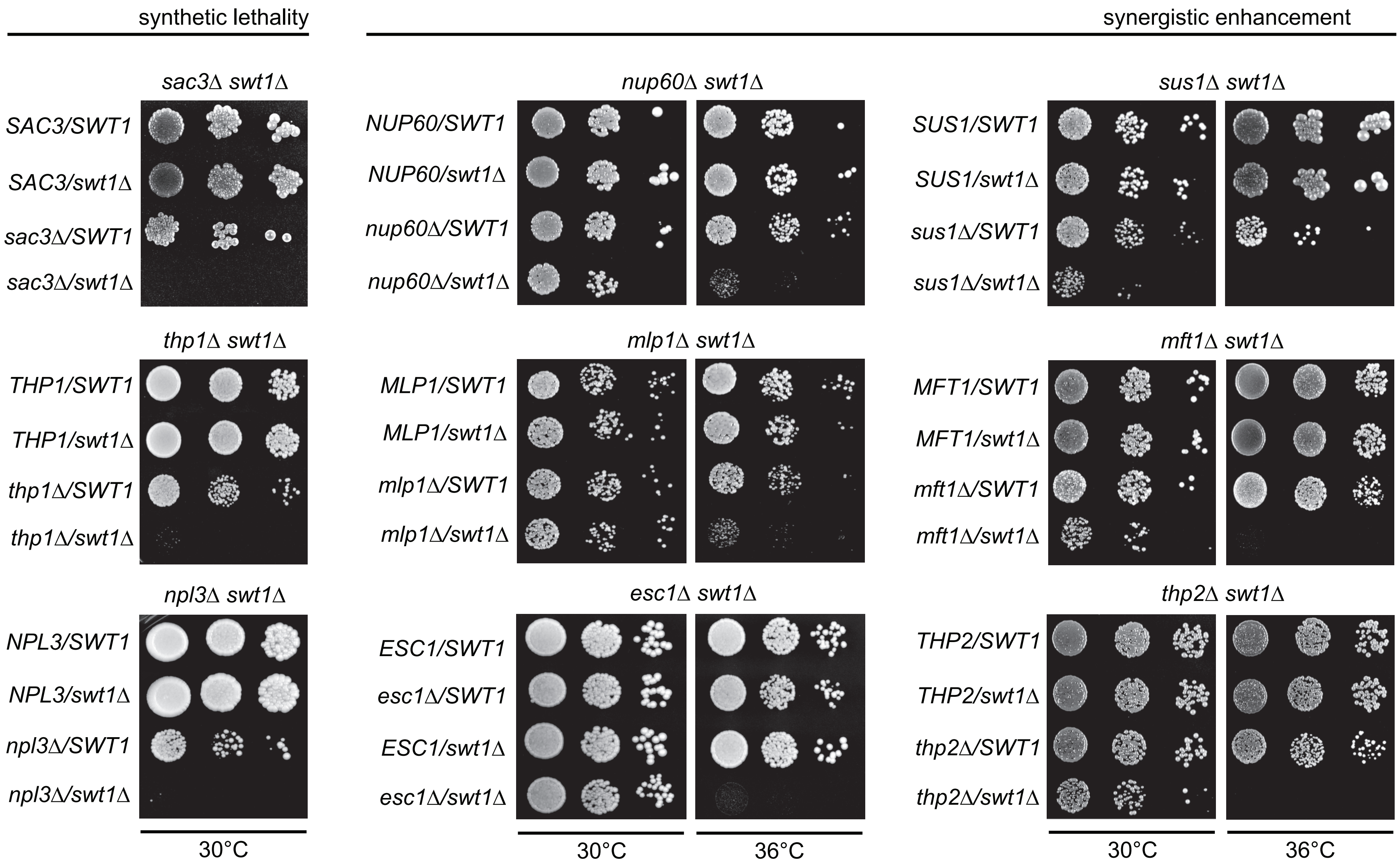

synthetic lethality
synergistic enhancement
sac3Δ swt1Δ
SAC3/SWT1
SAC3/swt1Δ
sac3Δ/SWT1
sac3Δ/swt1Δ
nup60Δ swt1Δ
NUP60/SWT1
NUP60/swt1Δ
nup60Δ/SWT1
nup60Δ/swt1Δ
sus1Δ swt1Δ
SUS1/SWT1
SUS1/swt1Δ
sus1Δ/SWT1
sus1Δ/swt1Δ
thp1Δ swt1Δ
THP1/SWT1
THP1/swt1Δ
thp1Δ/SWT1
thp1Δ/swt1Δ
mlp1Δ swt1Δ
MLP1/SWT1
MLP1/swt1Δ
mlp1Δ/SWT1
mlp1Δ/swt1Δ
mft1Δ swt1Δ
MFT1/SWT1
MFT1/swt1Δ
mft1Δ/SWT1
mft1Δ/swt1Δ
npl3Δ swt1Δ
NPL3/SWT1
NPL3/swt1Δ
npl3Δ/SWT1
npl3Δ/swt1Δ
esc1Δ swt1Δ
ESC1/SWT1
esc1Δ/SWT1
ESC1/swt1Δ
esc1Δ/swt1Δ
thp2Δ swt1Δ
THP2/SWT1
THP2/swt1Δ
thp2Δ/SWT1
thp2Δ/swt1Δ
30°C
30°C
36°C
30°C
36°C
