# Supplementary material for: An Endoribonuclease Functionally Linked to Perinuclear mRNP Quality Control Associates with the Nuclear Pore Complexes
Source: PLoS Biol. 2009 Jan 6;7(1):e1000008. doi: 10.1371/journal.pbio.1000008 (PMC2613419; doi:10.1371/journal.pbio.1000008)
Supplement: Supplementary file 2 [file pbio.1000008.sg002.pdf]

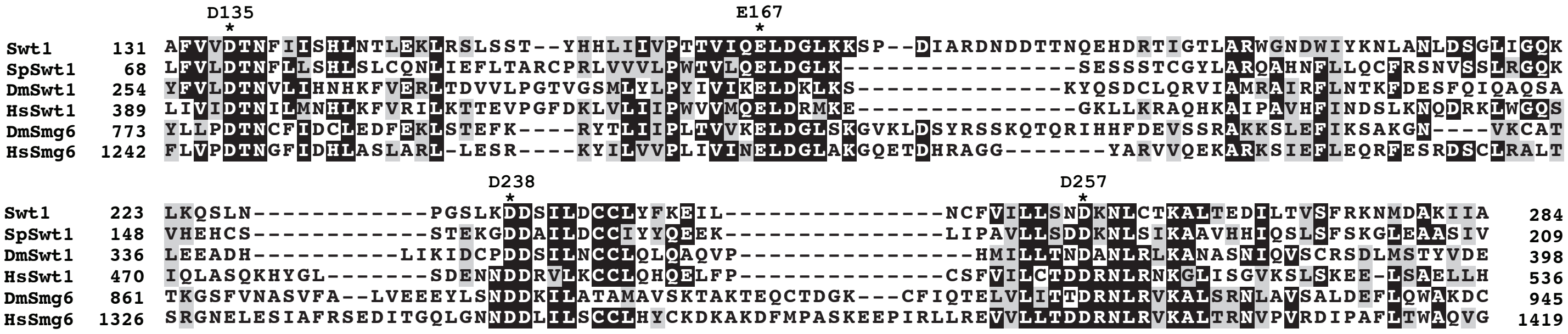
D135
E167
Swt1 131 AFVVDTNFIISHLNTLEKLRSLSST--YHHLIIVPTTVIQELDGLKKSP--DIARDNDDTTNQEHDRTIGTLARWGNDWIYKNLANLDSGLIGQK
SpSwt1 68 LFVLDTNFLLSHLSLCQNLIEFLTARCPRLVVVLPWTVLQELDGLK-----------------SESSSTCGYLARQAHNFLLQCFRSNVSSLRGQK
DmSwt1 254 YFVLDTNVLIHNHKFVERLTDVVLPGTVGSMLYLPYIVIKELDKLKS---------------KYQSDCLQRVIAMRAIRFLNTKFDESFQIQAQSA
HsSwt1 389 LIVIDTNILMNHLKFVRILKTTEVPGFDKLVLIIPWVVMQELDRMKE---------------GKLLKRAQHKAIPAVHFINDSLKNQDRKLWGQS
DmSmg6 773 YLLPDTNCFIDCLEDFEKLSTEFK----RYTLIIPLTVVKELDGLSKGVKLDSYRSSKQTQRIHHFDEVSSRAKKSLEFIKSAKGN----VKCAT
HsSmg6 1242 FLVPDTNGFIDHLASLARL-LESR----KYILVVPLIVINELDGLAKGQETDHRAGG-------YARVVQEKARKSIEFLEQRFESRDSCLRALT
D238
D257
Swt1 223 LKQSLN--------------PGSLKDDSILDCCLYFKEIL---------------NCFVILLSNDKNLCTKALTEDILTVSFRKNMDAKIIA 284
SpSwt1 148 VHEHCS--------------STEKGDDAILDCCIYYQEEK---------------LIPAVLLSDDKNLSIKAAVHHIQSLSFSKGLEAASIV 209
DmSwt1 336 LEEADH------------LIKIDCPDDSILNCCLQLQAQVP----------------HMILLTNDANLRLKANASNIQVSCRSDLMSTYVDE 398
HsSwt1 470 IQLASQKHYGL---------SDENNDDRVLKCCLQHQELFP--------------CSFVILCTDDRNLRNKGLISGVKSLSKEE-LSAELLH 536
DmSmg6 861 TKGSFVNASVFA--LVEEEYLSNDDKILATAMAVSKTAKTEQCTDGK---CFIQTELVLITTDRNLRVKALSRNLAVSALDEFLQWAKDC 945
HsSmg6 1326 SRGNELESIAFRSEDITGQLGNNDDLILSCCLHYCKDKAKDFMPASKEEPIRLLREVVLLTDDRNLRVKALTRNVPVRDIPAFLTWAQVG 1419
