# Supplementary material for: An Endoribonuclease Functionally Linked to Perinuclear mRNP Quality Control Associates with the Nuclear Pore Complexes
Source: PLoS Biol. 2009 Jan 6;7(1):e1000008. doi: 10.1371/journal.pbio.1000008 (PMC2613419; doi:10.1371/journal.pbio.1000008)
Supplement: Supplementary file 4 [file pbio.1000008.sg004.pdf]

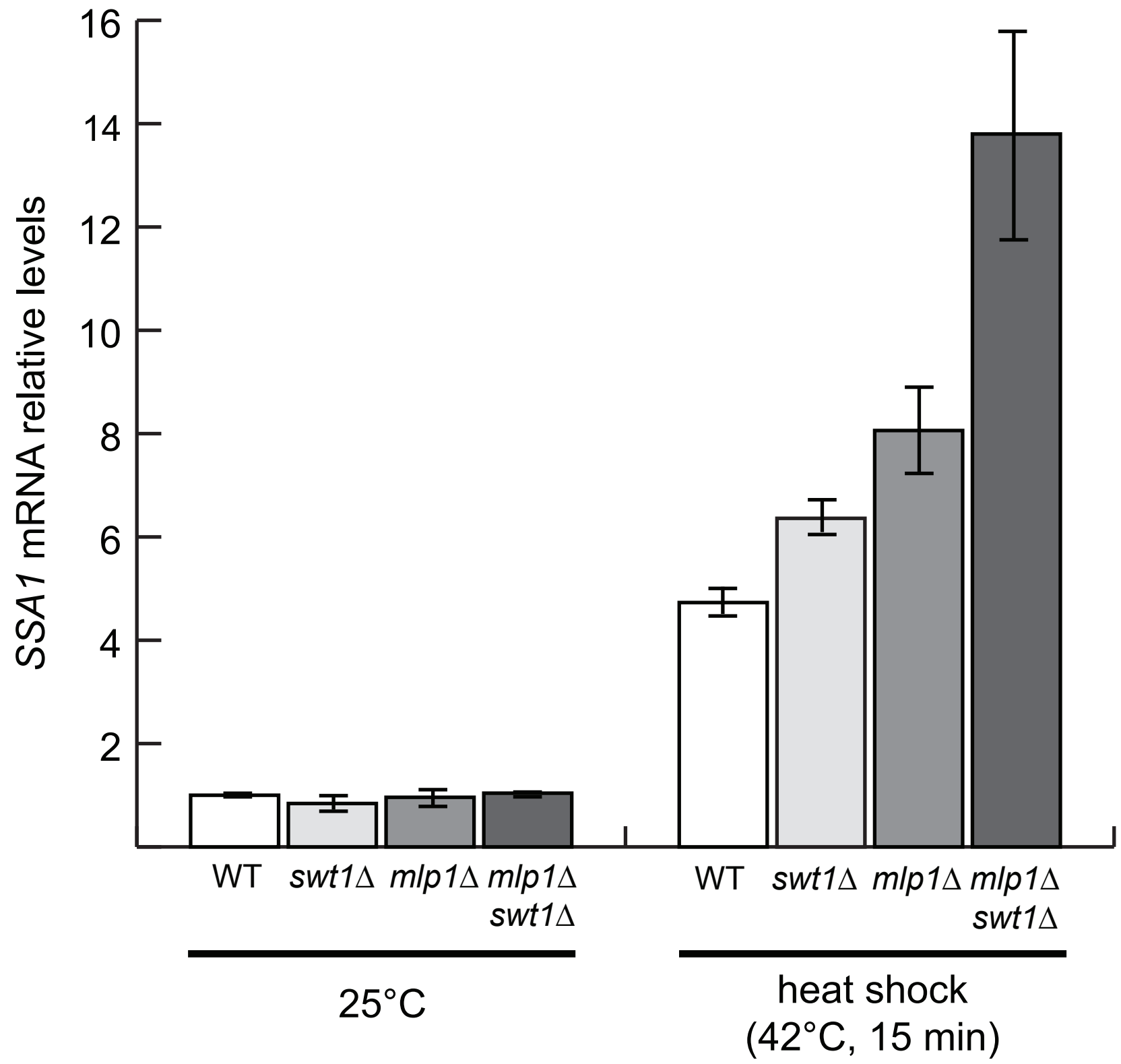

SSA1 mRNA relative levels
16
14
12
10
8
6
4
2
WT
swt1Δ
mlp1Δ
mlp1Δ swt1Δ
WT
swt1Δ
mlp1Δ
mlp1Δ swt1Δ
25°C
heat shock (42°C, 15 min)
