# Supplementary material for: An Endoribonuclease Functionally Linked to Perinuclear mRNP Quality Control Associates with the Nuclear Pore Complexes
Source: PLoS Biol. 2009 Jan 6;7(1):e1000008. doi: 10.1371/journal.pbio.1000008 (PMC2613419; doi:10.1371/journal.pbio.1000008)
Supplement: Supplementary file 5 [file pbio.1000008.sg005.pdf]

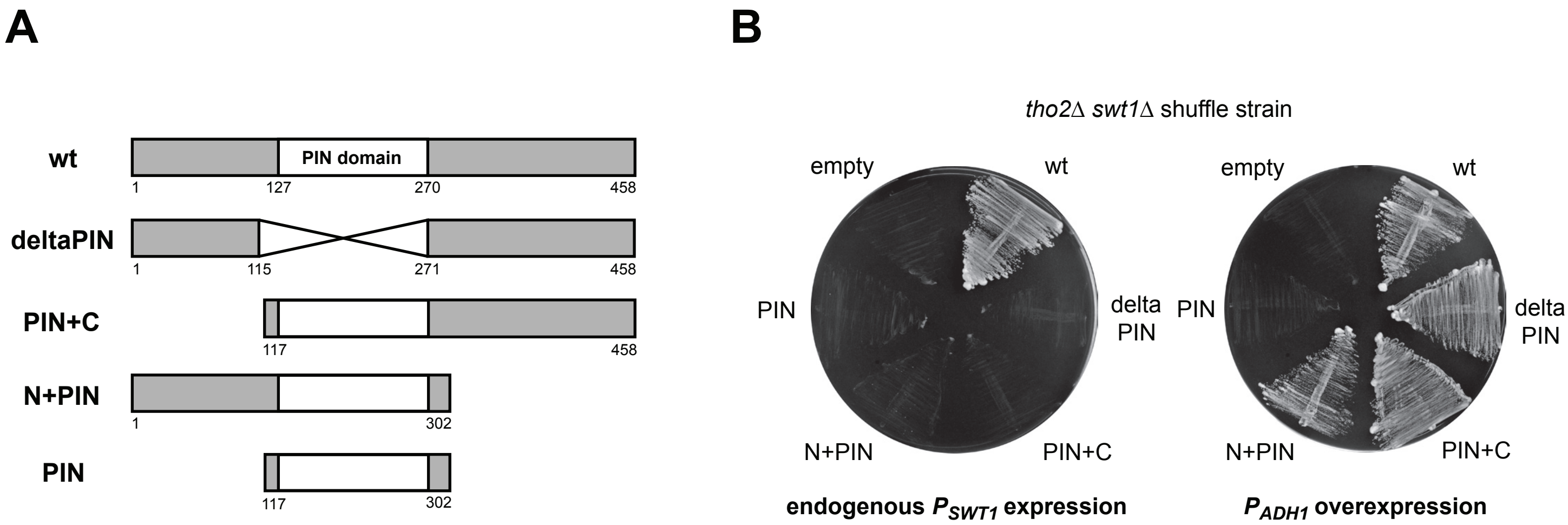
A
wt
PIN domain
1
127
270
458
deltaPIN
1
115
271
458
PIN+C
117
458
N+PIN
1
302
PIN
117
302
B
tho2Δ swt1Δ shuffle strain
empty
wt
PIN
delta PIN
N+PIN
PIN+C
endogenous P SWT1 expression
empty
wt
PIN
delta PIN
N+PIN
PIN+C
P ADH1 overexpression
