# Supplementary material for: An Endoribonuclease Functionally Linked to Perinuclear mRNP Quality Control Associates with the Nuclear Pore Complexes
Source: PLoS Biol. 2009 Jan 6;7(1):e1000008. doi: 10.1371/journal.pbio.1000008 (PMC2613419; doi:10.1371/journal.pbio.1000008)
Supplement: Supplementary file 6 [file pbio.1000008.sg006.pdf]

A

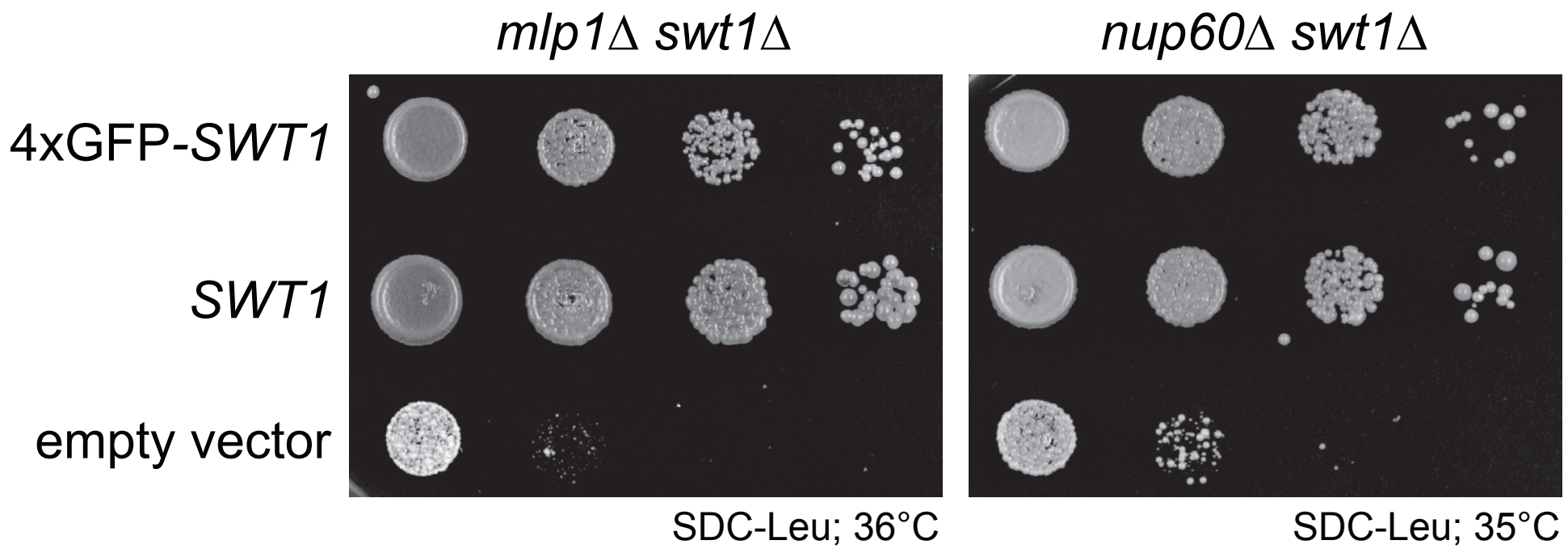
mlp1Δ swt1Δ
nup60Δ swt1Δ
4xGFP-SWT1
SWT1
empty vector
SDC-Leu; 36°C
SDC-Leu; 35°C

B

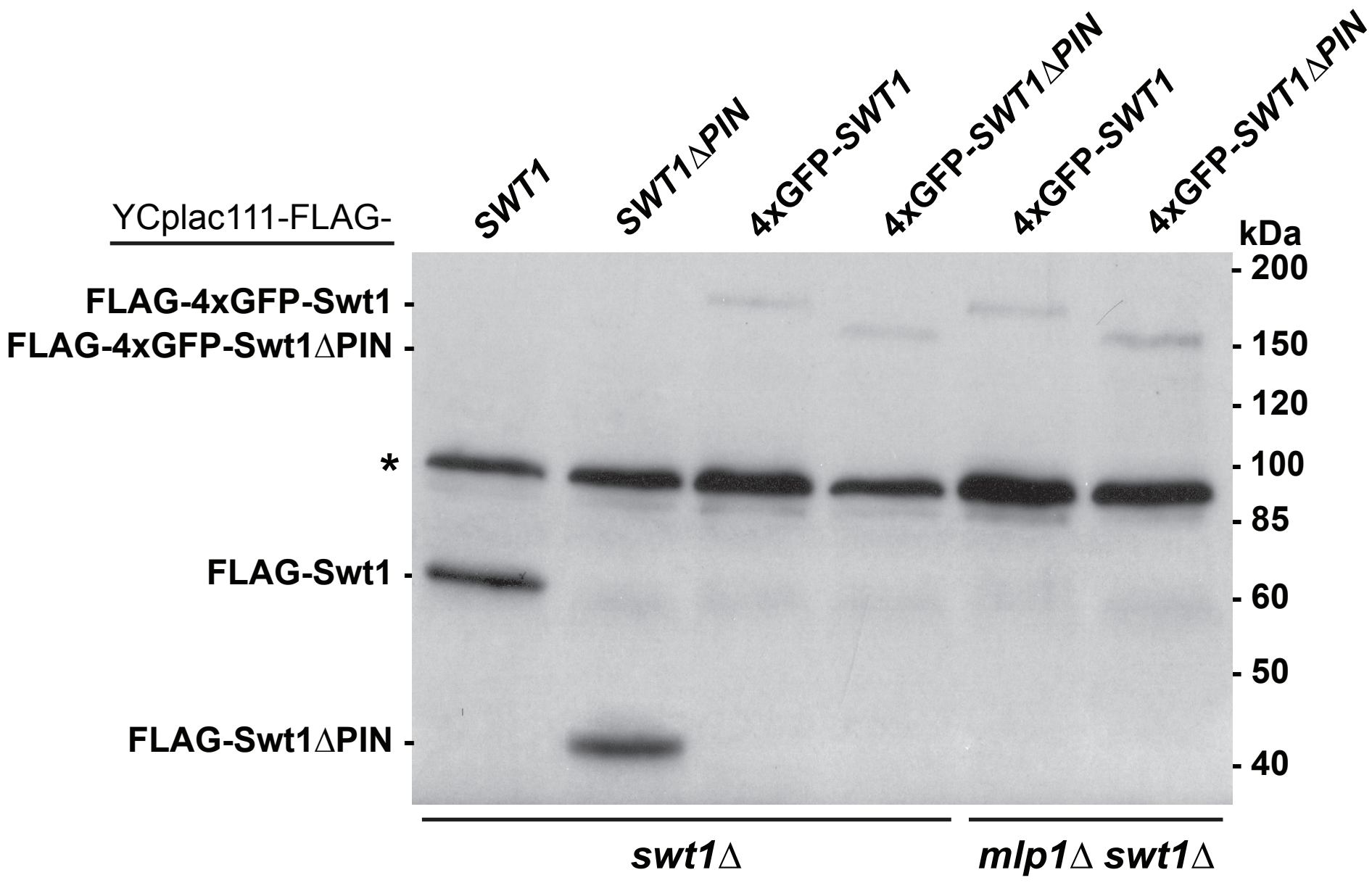
YCplac111-FLAG-
SWT1
SWT1ΔPIN
4xGFP-SWT1
4xGFP-SWT1ΔPIN
4xGFP-SWT1
4xGFP-SWT1ΔPIN
kDa
200
150
120
100
85
60
50
40
FLAG-4xGFP-Swt1
FLAG-4xGFP-Swt1ΔPIN
*
FLAG-Swt1
FLAG-Swt1ΔPIN
swt1Δ
mlp1Δ swt1Δ
