# Supplementary material for: An Endoribonuclease Functionally Linked to Perinuclear mRNP Quality Control Associates with the Nuclear Pore Complexes
Source: PLoS Biol. 2009 Jan 6;7(1):e1000008. doi: 10.1371/journal.pbio.1000008 (PMC2613419; doi:10.1371/journal.pbio.1000008)
Supplement: Supplementary file 7 [file pbio.1000008.st001.doc]

| **Table S1. Yeast strains used in this study** | |
| --- | --- |
| Strain | Genotype |
|  |  |
| RS453 | MATa/alpha *ade2-1 his3-11,15 leu2-3,112 trp1-1 ura3-52* |
| *sac3Δ* (*sl* screen) | MATa, *ade2 ade3 his3 leu2 trp1 ura3 sac3::KanMX*; pHT4465*Δ-SAC3* |
| *swt1Δ* | RS453, MATalpha *swt1::kanMX4* |
| *swt1Δ* (Euroscarf) | BY4742, MATa *his3Δ1 leu2Δ0 lys2Δ0 ura3Δ0* *swt1::kanMX4* |
| *sac3Δ swt1Δ* shuffle | MATa/alpha *ade2 his3 leu2 trp1 ura3 sac3::KAN swt1::kanMX4*; pRS316*-SAC3* |
| *thp1Δ swt1Δ* shuffle | MATa/alpha *his3 leu2 trp1 ura3 thp1::KAN swt1::kanMX4*; pRS316*-THP1* |
| *sus1Δ swt1Δ* | RS453, MATalpha *sus1::natNT2 swt1::kanMX4* |
| *nup60Δ swt1Δ* | MATa/alpha *ade2-1 his3-11,15 leu2-3,112 trp1-1 ura3-52 nup60::HIS5sp swt1::kanMX4* |
| *mlp1Δ swt1Δ* | RS453, MATa/alpha *mlp1::HIS3 mlp2::HIS3 swt1::kanMX4*; pRS314*-MLP2* |
| *mlp2Δ swt1Δ* | RS453, MATa/alpha *mlp1::HIS3 mlp2::HIS3 swt1::kanMX4*; pRS315*-MLP1* |
| *esc1Δ* | RS453, MATalpha *esc1::natNT2* |
| *esc1Δ swt1Δ* | RS453, MATalpha *esc1::natNT2 swt1::kanMX4* |
| *nup133Δ swt1Δ* | RS453, MATa/alpha *nup133::HIS3 swt1::kanMX4* |
| *mex67Δ swt1Δ* shuffle | RS453, MATa *mex67::HIS3* *swt1::natNT2*; pRS316-*MEX67* |
| *tho2Δ swt1Δ* shuffle | MATa/alpha *ade2-1 his3-11,15 leu2-3,112 trp1-1 ura3-52 tho2::KAN swt1::kanMX4*; pRS316*-SWT1* |
| *hpr1Δ swt1Δ* shuffle | MATa/alpha *ade2-1 his3-11,15 leu2-3,112 trp1-1 ura3-52 hpr1::HIS3 swt1::kanMX4*; pRS316*-HPR1* |
| *mft1Δ swt1Δ* | MATa/alpha *ade2-1 his3-11,15 leu2-3,112 trp1-1 ura3-52 mft1::KAN swt1::kanMX4* |
| *thp2Δ swt1Δ* | MATa/alpha *his3 leu2 trp1-1 ura3 thp2::kanMX4 swt1::kanMX4* |
| *sub2Δ swt1Δ* shuffle | MATa/alpha *leu2 trp1 ura3-52 sub2::kanMX4* *swt1::kanMX4*; pYCG-*SUB2* |
| *npl3Δ swt1Δ* shuffle | MATa/alpha *ade2 his3 leu2 trp1 ura3 npl3::kanMX4 swt1::kanMX4*; pRS316*-SWT1* |
| *yra1Δ swt1Δ* shuffle | RS453, MATa/alpha *yra1::HIS3 swt1::kanMX4*; pRS316-*YRA1* |
| *swt1Δ ubp8Δ* | RS453, MATalpha *swt1::kanMX4 ubp8::natNT2* |
| *swt1Δ cth1 cth2* | MATa/alpha *his3 leu2 ura3 cth1::kanMX4* *cth2::kanMX4* *swt1::kanMX4* |
|  |  |
| Further *swt1∆* double knockout/mutant strains were made by the mating of *swt1Δ* strain (see above) with the strains listed below, followed by the sporulation of the diploids, and the tetrade dissection and analysis. | |
|  |  |
| *dcp1-2* | MATa *his3 leu2 ura3 dcp1-2* |
| *dis3Δ* | MATa *his3 leu2 trp1 ura3 dis3* |
| *dob1-1* | MATa *ade2 his3 leu2 trp1 dob1-1* |
| *mtr3-1* | MATa *ura3 mtr3-1* |
| *nab2-1* | MATalpha *his3* *leu2 trp1 ura3 nab2::HIS3*; pLEU2*-nab2-1* |
| *nup1Δ* | RS453, MATalpha *nup1::HIS3* |
| *nup2Δ* | RS453, MATa *nup2::HIS3* |
| *nup116Δ* | RS453, MATa *nup116::HIS3* |
| *rat1-1* | MATalpha *his3 leu2 ura3 rat1-1* |
| *rna14-1* | MATa *ade2 his3 leu2 trp1 ura3 rna14-1* |
| *spt5-194* | MATa *his3Δ200 leu2Δ1 lys2-128Δ trp1Δ63 ura3-52 spt5-194* |
| *spt5-242* | MATa *his3Δ200 leu2Δ1 lys2-128Δ trp1Δ63 ura3-52 spt5-242* |
| *tho1Δ* | MATa *ade2 his3 leu2 trp1 ura3 tho1::TRP1* |
| *ulp1 ts* | MATa *his3Δ200 leu2-3,112::LEU2::ulp1-333 lys2-801 trp1-1 ura3-52*  *ulp1-1::his3::URA3* |
|  |  |
| *cbc1Δ*, *ccr4Δ*, *cdc73Δ*, *ctk1Δ, dom34Δ, gbp2Δ, lsm1Δ, mrt1Δ*, *nmd4Δ, paf1Δ*, *pan2Δ*, *pml1Δ*, *pml39Δ*, *rad26Δ*, *rpb4Δ*, *rrp6Δ*, *ski2Δ*, *ski7Δ, spt4Δ*, *trf4Δ*, *upf1Δ, upf2Δ, upf3Δ, xrn1Δ* deletion strains correspond to Euroscarf strain BY4741 (MATa *his3Δ1 leu2Δ0 met15Δ0 ura3Δ0*) with the disruption of respective gene by *kanMX4* cassette. | |
