# Supplementary material for: An Endoribonuclease Functionally Linked to Perinuclear mRNP Quality Control Associates with the Nuclear Pore Complexes
Source: PLoS Biol. 2009 Jan 6;7(1):e1000008. doi: 10.1371/journal.pbio.1000008 (PMC2613419; doi:10.1371/journal.pbio.1000008)
Supplement: Supplementary file 8 [file pbio.1000008.st002.doc]

| **Table S2. Plasmids used in this study** | |
| --- | --- |
| Name | Construction / Reference |
| pRS315-*SWT1*  pRS316-*SWT1* | The coding sequence of *SWT1* gene flanked by 300 bp and 200 bp of the 5' and 3' untranslated region, respectively, was PCR amplified using the genomic library plasmid complementing the *swt1-1* candidate of the *sac3Δ sl* screen as the template; and cloned into the *Xba*I-*Bam*HI sites of pRS315/pRS316. |
| YCplac111-NTAP-FLAG-*SWT1* | The coding and promotor sequences of *SWT1* gene were PCR amplified from pRS315-*SWT1* and ligated into the *Eco*RI-*Nco*I and *Xba*I-*Bam*HI sites of plasmid YCplac111-NTAP-FLAG-*RIX7* (a gift of D. Kressler), respectively. |
| YCplac111-FLAG-*SWT1* | The sequence of FLAG-*SWT1* fusion was PCR amplified from YCplac111-NTAP-FLAG-*SWT1* using the *Nco*I/*Xba*I site-containing primers and ligated into the same vector opened by *Nco*I and *Xba*I. |
| pADH111-FLAG-*SWT1* | The coding sequence of *SWT1* gene was released from YCplac111-NTAP-FLAG-*SWT1* by *Xba*I-*Bam*HI and ligated into plasmid YCplac111-*P_ADH1_*-NTAP-FLAG-*RIX7* (a gift of D. Kressler) opened by the same vay. Used for the expression of *SWT1* or *SWT1* D135N mutant from the *ADH1* promotor. |
| YCplac111-FLAG/-  pADH111-FLAG-  *SWT1ΔPIN* | The sequences coding for N- and C-terminus of Swt1 protein were PCR amplified using the *Xba*I/*Kpn*I and *Kpn*I/*Bam*HI sites-containing primers and ligated into *Xba*I-*Bam*HI sites of plasmids YCplac111-FLAG-*/*pADH111-FLAG-*SWT1.* |
| YCplac111-FLAG/ pADH111-FLAG-  *SWT1 deletion mutants* | The description of the cloning strategy of *SWT1* deletion mutants (Figure S5) into the indicated vectors is available on the request. |
| YCplac111-*SWT1* D135N | The D135N mutation of *SWT1* gene was generated by the fusion PCR (see Materials and methods) using pRS315-*SWT1* as the template. The resulting PCR product was ligated into the *Nco*I and *Xba*I sites of YCplac111-FLAG-*SWT1*. |
| pCMha191-*SWT1* | The *SWT1* ORF was PCR amplified from pRS315-*SWT1* using BglII-containing primers and ligated into the BamHI site of pCMha191 (Boyer et al., 2004). |
| YEplac112Gal*-SWT1* | HA*-SWT1-T_CYC1_* fragment of pCMha191-*SWT1* was amplified by PCR and cloned into the *Xba*I-*Sph*I sites of YEplac112Gal. |
| YEplac112Gal*-SWT1*  D135N | The part of *SWT1* ORF was cut out from YCplac111-*SWT1* D135N by *Nhe*I-*Bsa*BI and inserted into the similarly opened YEplac112Gal*-SWT1*. |
| pNOPGFPA1A-*SWT1*  pNOPGFPA1L-*SWT1* | The coding sequence of *SWT1* was PCR amplified from the genomic library plasmid (see above), cloned into the *Nco*I-*Bam*HI sites of pNOPPATA1L, then  cut and religated as the *Pst*I fragment into pNOPGFPA1L. |
| pNOPGFPA1L-*SWT1ΔPIN* | The sequence of *NOP1* promotor and *GFP* moiety was PCR amplified from vector pNOPGFPA1L by *Eco*RI/*Ole*I-sites containing primers and inserted into the *Eco*RI-*Ole*I opened plasmid YCplac111-FLAG-*SWT1 deltaPIN.* |
| YCplac111-FLAG-4xGFP-*SWT1/*  *SWT1ΔPIN* | The sequence of four EGFP modules was PCR amplified from plasmid pmEGFP5-N1 (constructed by N. Daigle, A. Bancaud, and J. Ellenberg; EMBL Heidelberg) and inserted into the XbaI site of plasmids YCplac111-FLAG-*SWT1/*YCplac111-FLAG*-SWTΔPIN.* |
| pET24d-GST-TEV  -*SWT1*-HIS | The coding sequence of *SWT1* was PCR amplified from the genomic library plasmid (see above) and cloned into the *Bam*HI-*Xho*I sites of pET24d-GST-TEV. Used for the *E.coli* expression of Swt1 with aim to generate anti-Swt1 sera. |
| pHT4465*Δ-SAC3* | Hurt Lab plasmid collection |
| pRS315-*SAC3*  pRS316-*SAC3* | Fischer et al. (2002) EMBO J. 21: 5843-5852. |
| pRS315*-THP1*  pRS316-*THP1* | The genomic fragment of *THP1* was cut out from plasmid pRS416*-THP1* (Fischer et al*.*, 2002) by *Not*I and cloned into the same site of pRS315. |
| pRS315-*SAC3*  pRS316-*SAC3* | Fischer et al. (2002) EMBO J. 21: 5843-5852. |
| pRS315-*SUS1* | Rodriguez-Navarro et al. (2004) Cell 116: 75-86. |
| pRS315*-THO2* | Piruat JI, Aguilera A (1998) EMBO J 17: 4859-4872. |
| pUN100*-HPR1*  pRS316*-HPR1* | Strässer et al. (2002) Nature 417: 304-307. |
| pRS315*-MFT1* | Strässer et al. (2002) Nature 417: 304-307. |
| pRS315*-THP2* | Strässer et al. (2002) Nature 417: 304-307. |
| YCp50-*NPL3* | Lee et al. (1996) Genes Dev 10: 1233-1246. |
| pRS314-*sub2-85* | Strässer K, Hurt E (2001) Nature 413: 648-652. |
| pRS314-*yra1-ΔN* | Strässer K, Hurt E (2001) Nature 413: 648-652. |
| pRS314-*yra1-ΔRRM* | Strässer K, Hurt E (2001) Nature 413: 648-652. |
| pUN100*-mex67-5* | Segref et al. (1997) EMBO J 16: 3256-3271. |
| pUN100*-NUP60* | Fischer et al. (2002) EMBO J. 21: 5843-5852. |
| pRS315*-MLP1* | Galy et al*.* (2004) Cell 116: 63-73. |
| pRS314*-MLP1* | The genomic fragment of *MLP1* was cut out from plasmid pRS315*-MLP1* (Galy et al*.*, 2004) by *Bam*HI*-Sal*I and cloned into the same sites of pRS314. |
| pRS316*-MLP2* | Hurt Lab plasmid collection (constructed by K. Strässer). |
| pRS316-*NUP133* | Doye et al. (1994) EMBO J 13: 6062-6075. |
| pJCR1, pJCR51,  pmut5’SS, pmutBP,  pLGSD5 | Legrain P, Rosbash M (1989) Cell 57: 573-583;  Rain JC, Legrain P (1997) EMBO J. 16: 1759-1771;  Galy et al*.* (2004) Cell 116: 63-73 and references therein. |
| pRS314-*NUP120*-mCherry | Hurt Lab plasmid collection (constructed by P. Stelter). |
