# Supplementary material for: An Endoribonuclease Functionally Linked to Perinuclear mRNP Quality Control Associates with the Nuclear Pore Complexes
Source: PLoS Biol. 2009 Jan 6;7(1):e1000008. doi: 10.1371/journal.pbio.1000008 (PMC2613419; doi:10.1371/journal.pbio.1000008)
Supplement: Supplementary file 9 [file pbio.1000008.st003.doc]

| **Table S3. Real-time RT-PCR oligonucleotides used in this study** | |
| --- | --- |
| Name | Sequence |
| *SSA1* forward  *SSA1* reverse  *SSA1* probe | 5’-GGTATCGATTTCTACACTTCCATCAC-3’  5’-CAACTGGGTCCAAAGTAGATCTGA-3’  5’-6-FAM-AGAGCCAGATTCGAAGAATTGTGTGCTGA-TAMRA-3’ |
| *RPB4* forward  *RPB4* reverse  *RPB4* probe | 5’-AAGCACTGGGTTACATCCTTTTG-3’  5’-TCTTTGCTTCATCAGCTGTGTCA-3’  5’-6-FAM-AGTGGCGCAACTAGGTTCTTTGGCCT-TAMRA-3’ |
| *lacZ* forward  *lacZ* reverse  *lacZ* probe | 5’-ATCAGGATATGTGGCGGATGA-3’  5’-TGATTTGTGTAGTCGGTTTATGCA-3’  5’-6-FAM-CGGCATTTTCCGTGACGTCTCGTT-TAMRA-3’ |
| *GAL1* forward  *GAL1* reverse  *GAL1* probe | 5’-TGAAGAGTCTCTCGCCAATAAGAA-3’  5’-TCGCGAGAACAATTCAAGGA-3’  5’-6-FAM-AGGGCTTTAGTGTTGACGATGTCGCACA-TAMRA-3’ |
